# The population health effects from 5G: Controlling the narrative

**DOI:** 10.3389/fpubh.2022.1082031

**Published:** 2022-12-19

**Authors:** Frank de Vocht, Patricia Albers

**Affiliations:** Population Health Sciences, Bristol Medical School, University of Bristol, Bristol, United Kingdom

**Keywords:** 5G, radiofrequency (RF), mobile phones, cellphones, bias, review, conflicts of interest (COI)

## Introduction

The development and implementation of the fifth-generation wireless technology (5G) are currently ongoing and have largely been met with enthusiasm from the telecommunication industry, applications industries, national governments, and the public. However, 5G has also been met with resistance from anti-5G campaigning organizations supported by pockets of the general public. Concerns relate to the perception that 5G might increase total exposure to radiofrequency (RF) radiation, with further concerns around the fact that in addition to the frequency bands used in 3G and 4G, 5G will (and in some places already does) also use frequencies of >6 GHz including a new ~ 30–300 GHz “high band” with wavelengths from 10 to 1 mm [millimeter waves (MMWs)] (1). Further concerns relate to the use of multiple-input multiple-output (MIMO) technologies and beamforming, and to the implications on infrastructure as 5G requires many additional new small cells. A cursory read of popular and social media provides interesting reading and illustrates how different interpretations of the same information can result in widely varying interpretations, not least compounded by 5G-related conspiracy theories (2). Competing narratives around 5G are also described around geopolitical debates (3). Ideally, the peer-reviewed evidence synthesis literature should be free of these and other non-scientific influences, but in practice, this is rarely, if ever, the case. To explore the narrative that formed the basis for the evaluation of health risks in the peer-reviewed scientific literature, the publications on the topic published during the first critical period of discussion are briefly reviewed and discussed.

## Methods

PubMed, Ovid Medline, and Web of Science databases of peer-reviewed literature were searched for reviews, commentaries, and opinion articles related to 5G and health. Inclusion was limited to these publications as these provide overviews of the evidence and/or initiate, drive, or direct the scientific debate, and primary research studies were excluded. Only publications in English language were included, and an *a priori* cutoff of the first 3 years from the first publication was assumed to describe the initiation and direction of the debate. Included articles were ranked based on the month and year of online publication (often “ahead of print”) to provide a chronological timeline of when information would have become available. Articles were assigned as “industry” or “activism” depending on whether the articles report links between the authors and either industry or campaigning organizations related to 5G in particular or mobile phones more broadly, or as “independent” otherwise. In case no such links were reported, a basic internet search was performed to identify unreported links.

## Results

An overview of the 15 articles included in this review is provided in Table 1. The set of articles covered the period of 2018–2021, thus providing an overview of the first 3 years of publications on 5G and health.

**Table 1:** Overview of included publications.

**No**.	**Year and month of first (online) publication**	**First** **author**	**Funding**	**Reported** **CoI**	**Publication** **type**	**Independent/** **industry/** **activism** ^1^	**Detail of methodology for evidence synthesis (A–C)^2^**	**Assessment of study quality**	**Reported biological or health effects from 5G-related RF^3^**	**Policy recommen** **dation**
1	2018 (Feb)	Di Ciaula	Not provided	None declared	Systematic-style review	Activism	B	No	+	Yes
2	2018 (April)	Russell	Reported no external funding	None declared	Narrative review	Activism	B	No	+	Yes
3	2018 (August)	McClelland	Not provided	Not provided	Commentary	Independent	C	No	+	Yes
4	2019 (August)	Miller et al.	Not provided	One CoI (legal counsel)	Narrative review	Activism	C	No	+	Yes
5	2019 (September)	Simkó	Industry	None declared	Systematic-style review	Industry	A	Yes	+/–	No
6	2020 (January)	Hardell	Reported no external funding	None declared	Commentary	Activism	C	No	+	Yes
7	2020 (January)	Kostoff	Not provided	None declared	Narrative review	Independent/ Activism	C	No	+	No
8	2020 (June)	Bushberg	Not provided	All (industry)	Narrative review	Industry	C	Yes	-	No
10	2020 (July)	Hardell	Reported no external funding	None declared	Commentary	Activism	C	No	+	Yes
9	2020 (August)	Leszczynski	Reported no external funding	None declared	Systematic-style review	Independent	A	Yes	+/–	Yes
11	2021 (January)	Frank	Reported no external funding	None declared	Essay	Activism	C	No	+	Yes
12	2021 (March)	Karipidis	Governmental and Research Council	None declared	Systematic-style review	Andependent	A	Yes	–	No
13	2021 (March)	Wood	Governmental and Research Council	None declared	Meta-analysis	Independent	A	Yes	–	No
14	2021 (March)	Jargin	Reported no external funding	None declared	Letter to the editor	Independent	C	No	–	No
15	2021 (June)	Hardell	Not provided	None declared	Opinion review	Activism	C	No	+	Yes

1 These crude labels are used for high-level comparison. “Activism” is used where there are links to NGOs with an aim to influence radiation policy.

2 Detail provided classified as A (methods described), B (limited, insufficient information provided), C (not reported).

3 Included publications either reported adverse impacts on all covered health and biological effects (+) or reported no health or biological effects (–), with few reporting adverse impacts for only some of the covered effects (+/–).

The first review was published in February 2018 by Di Ciaula (4) and was based on a systematic search of epidemiological, *in vivo*, and *in vitro* studies identified in the PubMed database. Di Ciaula reported no funding or conflict of interest (CoI), but an internet search identified membership of the International Society of Doctors for Environment (ISDE), which published a 5G appeal for a moratorium on the development of 5G (https://www.isde.org/5G_appeal.pdf). Di Ciaula discussed the evidence for cancer, reproductive effects, neurologic effects, and microbiological effects and specifically addressed evidence in relation to MMWs. No formal assessment of the quality of the studies was included, and the author concluded that “[the evidence] clearly point to the existence of multi-level interactions between high-frequency EMF and biological systems, and to the possibility of oncologic and non-oncologic (mainly reproductive, metabolic, neurologic, microbiologic) effects” and further raises concerns regarding the increased susceptibility of children. The main aim of the review was to provide the rationale to invoke the precautionary principle, which is mentioned both in the Conclusion section and Abstract.

Russell published a similar review in April 2018 (5). Despite being the Executive Director of Physicians for Safe Technology, the author reported no affiliation, funding, or CoI. Russell does acknowledge support from Smernoff and Moskowitz; an internet search identifies the latter as being on the Advisory Board of Physicians for Safe Technology as well as being an advisor to the International EMF Scientist Appeal (and its spokesperson for the United States). The review reported effects on cancer, dermal effects, ocular effects, effects on reproduction and neurology, microbiological effects, and effects on the immune system. It further reports specific effects from MMWs, electrohypersensitivity [or, more accurately, idiopathic environmental intolerance attributed to electromagnetic fields (IEI-EMF)], and effects on children, and discusses how industry bias has obscured these facts. Scientific uncertainty is only mentioned in passing and is largely attributed to industry distortion. Russell concludes that “current radiofrequency radiation wavelengths we are exposed to appear to act as a toxin to biological systems” and “although 5G technology may have many unimagined uses and benefits, it is also increasingly clear that significant negative consequences to human health and ecosystems could occur if it is widely adopted.” It further makes specific policy recommendations that “public health regulations need to be updated to match appropriate independent science with the adoption of biologically based exposure standards prior to further deployment of 4G or 5G technology” and that “a moratorium on the deployment of 5G is warranted, along with the development of independent health and environmental advisory boards that include independent scientists who research biological effects and exposure levels of radiofrequency radiation.”

McClelland and Jaboin, who do not seem to have published on the topic of mobile phones and health before, published a commentary in August 2018 (6). They reported no CoIs, the commentary was supported by a few references to *in vivo* studies, and the sole aim of the commentary was to bring a 5G moratorium to the attention of the journal's readership.

Miller et al. published their review on August 2019 (7). The manuscript was initially developed as a Position Statement of the International Network for Epidemiology in Policy (INEP), but after its board voted to abandon its involvement, the authors decided to publish it regardless. They reported affiliations to universities as well as the campaigning organizations the Environmental Health Trust and the Environment and Cancer Research Foundation, but did not, for example, report their involvement in the Physician's Health Initiative for Radiation and Environment (PHIRE) (Miller, Hardell, Davis) and Oceania Radiofrequency Scientific Advisory Association (ORSAA) (Hardell, Morgan, Davis). No information is provided on the methodology of this narrative review, and no quality assessment of included references is conducted, but scientific uncertainty is discussed. Carcinogenic and reproductive effects are reported as a specific susceptibility of children to RF. Particularly in relation to 5G, skin effects, oxidative stress, altered gene expression, immune function, and other biological endpoints are mentioned. The authors make several policy recommendations, but not specifically in relation to 5G.

In September 2019, Simkó and Mattsson published a pragmatic review of *in vivo* and *in vitro* evidence for health and biological effects in relation to 6 to 100 GHz frequency range (8). Both authors were from SciProof International and reported that their review was funded by Deutsche Telekom Technik GmbH. Although described in opaque language, the review seems to be based on a systematic approach to evidence synthesis and includes an assessment of study quality. Scientific uncertainty is discussed in detail, and the authors conclude that “regarding the health effects of 6–100 GHz at power densities not exceeding the exposure guidelines, the studies provide no clear evidence due to contradictory information from the *in vivo* and *in vitro* investigations.” They further highlight that “regarding the quality of the presented studies, a few studies fulfill the minimal quality criteria to allow any further conclusions.”

Hardell and Nyberg published a commentary in January 2020 (9). Both reported university affiliations and reported that neither funding was received for the work nor do they report any CoIs. However, in addition to unreported associations already mentioned above, it has also been documented that Hardell has previously received direct industry funding as well as funding from pressure groups, while he has also acted as an expert witness for the plaintiff in hearings around brain tumors and mobile phones (10). He is the spokesperson for the International EMF Scientist Appeal for Sweden and also runs a charity, the Environment and Cancer Research Foundation, which accepts direct donations and is heavily involved in appeals. The commentary includes several strong claims, including that “RF radiation may now be classified as a human carcinogen, Group 1” and that “experience with the EU, and the governments of the Nordic countries suggest that the majority of decision-makers are scientifically uninformed on health risks from RF radiation”, and interestingly and without basis that “they [the EU and governments of Nordic countries] seem to be uninterested to being informed by scientists representing the majority of the scientific community.”

In January 2020, there was also the publication of a review of health effects of 5G under real-life conditions by Kostoff et al. (11). They reported university affiliations and declared that neither external funding was received for the work nor any CoIs. However, an internet search identified that Héroux is the spokesperson for the International EMF Scientists Appeal for Canada. There is no assessment of study quality or scientific uncertainty. They mentioned that industry influence is the cause of the lack of consensus on health effects of mobile phones. The authors claimed that “there is a large body of data from laboratory and epidemiological studies showing that previous and present generations of wireless networking technology have significant adverse health impacts”, and that, with respect to 5G specifically, “superimposing 5G radiation on an already imbedded toxic wireless radiation environment will exacerbate the adverse health effects shown to exist.”

An information statement from the IEEE Committee on Man and Radiation (COMAR) was published in relation to health and safety issues concerning the exposure of the general public to electromagnetic energy from 5G wireless communication networks in June 2020 (1). All authors report industry CoIs. The main focus of the review relates to RF exposures from 5G, but some discussion specifically on potential biological and health effects of MMWs is included. Study quality is discussed in detail, including the varying quality of narrative reviews [including (4)], and research gaps regarding the bioeffects of MMWs are highlighted. The authors refer back to (8) for a discussion on bioeffects and conclude that “… while we acknowledge gaps in the scientific literature, particularly for exposures at MMW frequencies, the likelihood of yet unknown health hazards at exposure levels within current exposure limits is considered to be very low, if they exist at all.”

Hardell contributed a second commentary in this period, with Carlberg as co-author (12). In this commentary, they reported the Environmental and Cancer Research Foundation as their affiliation, but declared neither CoI nor any external funding for the work. Also, the authors discussed the involvement of certain experts in various committees related to RF health and safety in the EU and internationally and the influence of industry. In addition, they mentioned effects of RF exposure, including 5G, on cancer, reproduction, and neurology; effects on the immune system; and microbiological effects, and also mentioned the susceptibility of children to RF. The claim that “the IARC Category should be upgraded from Group 2B to Group 1, a human carcinogen” is re-iterated, referencing Hardell's earlier contribution as the basis for this claim (9). Hardell and Carlberg highlighted the appeal for a 5G moratorium sent to the EU in 2017.

Leszczynski published a review on the physiological effects of MMWs on the skin and skin cells in August 2020 (13). He reports a university affiliation, neither external funding for the work nor CoI. Leszczynski conducted a systematic review of several databases for studies of >6 GHz. The quality and uncertainty of the available evidence are specifically discussed, and he concludes that “this evidence is currently insufficient to claim that any effects have been proven or disproven”. Leszczynski addresses policy and argues that “deployment for industrial use should be the first, but the further broader deployment for the non-industrial use should preferably await for the results of the biomedical research”.

Frank published an essay on 5G and the precautionary principle in January 2021 (14). He declares neither external funding nor CoI. He is, however, a member of the PHIRE team. Frank has no previous track record in radiation epidemiology, but he has reviewed the evidence and provided support for the work by Miller et al. (7). He concluded that the precautionary principle should be applied and recommended a moratorium on 5G development.

A team from the Swinburne University of Technology and the Australian Radiation Protection and Nuclear Safety Agency (ARPANSA) published two studies in March 2021: a comprehensive review of the literature for experimental studies of bioeffects of RF fields between 6 and 300 GHz and a complementary meta-analysis (15, 16). The authors reported Australian government and National Health and Medical Research Council funding, but no CoIs. Of relevance is that Karipidis is a member of the International Commission on Non-Ionizing Radiation Protection (ICRNIRP). The included studies in these publications were identified in a systematic literature search, and the authors have explicitly discussed study quality. They concluded that many studies have low-quality methods and that experimental data do not provide evidence that low-level MMWs are associated with biological effects relevant to human health.

Jargin published a letter to the editor in March 2021 (17) in which he has argued that various publications claiming there are health harms related to 5G published by interest groups overestimate any health risks from RF-EMF to hamper the technological advancement of developed nations. He further argued that excessive restrictions would only be unfavorable for the economy and add difficulties to daily life. As such, it advocates a policy recommendation of no action. He has reported neither external funding for the work nor any CoI.

Hardell also contributed a third publication (18). In this opinion piece/review, Hardell argued that evaluations by the Health Council of the Netherlands, the WHO, ICNIRP, and the Swedish Radiation Safety Authority are not impartial and that a moratorium on the implementation of 5G is urgently required. He has reported both university and foundation affiliations, but has reported neither external funding nor any of the above identified CoI.

## Discussion

This chronological overview of the publications published during the initial critical phase of discussions around 5G and health leads to the interesting observation that publications by authors with links to anti-5G campaigning organizations dominated the early phase in which adverse effects related to 5G were discussed. Over half of the 15 publications had links to such organizations in the initial 3-year period covered here. Such patterns of efforts to control the narrative during critical periods have been studied elsewhere, for example, in the sugar-sweetened beverage research (19); although in this example, the opposite pattern was observed in which the contribution of industry-related studies was high at the start and decreased significantly with time.

With the increasing contribution from independent and industry-linked authors over the covered time period, the narrative shifts from the exclusive reporting of increased risks of all biological or health effects covered to predominantly descriptions of mixed results and conclusions not supporting increased risks. This difference in the interpretation of the same evidence depending on the affiliation in RF research has been mentioned previously, specifically in relation to the funding source of primary studies (20, 21), but the current overview is indicative of a similar pattern in other types of peer-reviewed publications. Reviews from independent and industry-linked authors were systematic-style reviews, rather than narrative reviews, and were of higher methodological quality because they based their inferences on a more systematic approach to the identification of relevant literature and also explicitly included some forms of assessment of the quality of these studies. They also had a narrower aim in terms of exposures or health outcomes, which will have facilitated a more systematic approach. There is evidence from various industries, including the telecommunications industry (20, 21), of a correlation between industry funding of research and null findings. However, there is much less discussion of its mirror image: the phenomenon that independently funded studies may be biased if the authors have strong *a priori* beliefs about the question under study. This “white hat bias” is observable in the literature as selective referencing and the acceptance of a lower standard of scientific evidence for studies supporting the authors' beliefs (22), and was first explored in obesity research (23, 24). The non-systematic inclusion of references (or “cherry picking”) and lack of explicit assessment of study quality observed in the publications in the current work were most prominent in the narrative reviews by authors with links to campaigning organizations and likely will have resulted in biased inferences. Importantly, since these publications made up most of the earliest publications during the critical window, these inferences will have disproportionally influenced the narrative. Given that all of these articles had the specific aim to influence policy and, in most cases, advocated for a moratorium on 5G, this provides further support for the presence of “white hat bias” influencing the initial peer-reviewed and, through that, lay literature.

Given the observed differences between publications by authors with links to campaigning organizations and those with industry-linked or independent authors, the reporting of CoI becomes more important. Direct industry funding and other financial CoIs are generally considered the main sources of potential bias, and these were reported by the publications with links to industry (either as a CoI or as a funding source) and by one of the papers with links to activism. However, no other financial CoIs were reported; for example, it is recorded that Hardell, who has contributed three publications in this critical time period, has previously received direct industry funding as well as funding from pressure groups, while he has also acted as an expert witness for the plaintiff in hearings around brain tumors and mobile phones (10). Importantly, industry and other financial CoIs are not the only potential source of CoI bias (25), and a variety of non-financial CoIs have been described, for instance, originating from particular concerns, ideals, and predilections (26). Membership of campaigning organizations or their advisory or expert boards would, presumably, constitute such non-financial CoIs and, therefore, should have been reported. Despite internet searches by the authors identifying quite a number of such CoIs, only a few of these were reported by the authors (or could be inferred from affiliations). Likewise, the membership of national or international expert organizations constitutes non-financial CoIs that ideally should have been reported, and Karipidis' membership of ICNIRP is relevant in the context of these publications.

Although the discussed timeline of publications highlights some interesting trends and areas of concern, this work has a number of limitations. Although the selected manuscripts were identified through a systematic search, it was not a systematic review of the literature, and publications that did not specifically mention 5G in the title, abstract, or keywords might have been missed. Furthermore, the search was also limited to publications in English language. Although the wider debate about health effects of 5G is much larger and also includes gray literature, popular, and social media, these were not included in this overview. It would be an interesting future exercise to evaluate similar trends in these media. Although several non-reported CoIs were identified, these were identified following cursory internet searches only and do not constitute an exhaustive list. It is likely that a more thorough systematic search would reveal additional links not reported here. It is also possible that some such CoIs did not exist yet at the time of publication.

In conclusion, the discussion around 5G as a significant human health risk in the peer-reviewed literature was initially largely driven by authors from, or with links to, various campaigning organizations and linked publications directly to appeals for a moratorium on 5G. Commentaries and letters are personal opinions and are rarely based upon a methodological appraisal of the evidence, but the narrative of the initial period covered in the current review, relied mostly on reviews of lower methodological quality compared, with the subsequently published reviews by independent researchers and researchers with links to industry. It is likely that articles in the popular media, therefore, were influenced more heavily by the initial advocacy publications than by the later higher quality contributions. Importantly, there is no clear answer (yet) whether the resulting narrative from the peer-reviewed literature describes an overestimation of risks as a result of articles with links to campaigning organizations, or whether later contributions from authors with links to industry, and possibly most independent authors, at the latter stages of the critical window describe an underestimation of true causal associations, or whether their combined evaluation will inform future evidence synthesis closer to “the truth”. It is, however, well established that not including explicit evaluation of the quality of studies included in evidence synthesis, and which was most evident in publications classified as “activism”, makes such reviews more susceptible to biased inferences. In addition to issues related to controlling the narrative and the impact of “white hat bias”, the current work further describes undisclosed non-financial CoIs that are likely to have influenced the interpretation of evidence. This was also observed particularly for those publications associated with campaigning organizations. The narrative around 5G and potential human health effects should be interpreted through this lens, in particular because many of the authors with links to various campaigning organizations in this article (Hardell, Héroux, Miller, and Moskowitz) as well as others who published works after the covered period have recently joined up formally in a new advocacy group ICBE-EMF (27).

## Author contributions

FdV conceived of the study and wrote the first version of the manuscript. FdV and PA conducted the analyses. All authors contributed to the article and approved the submitted version.
